# Effect of Health Status and Heat-Induced Inactivation on the Proteomic Profile of Plasma Rich in Growth Factors Obtained from Donors with Chronic Inflammatory Skin Conditions

**DOI:** 10.3390/biom14070763

**Published:** 2024-06-26

**Authors:** Eduardo Anitua, Roberto Tierno, Mikel Azkargorta, Félix Elortza, Mohammad H. Alkhraisat

**Affiliations:** 1 University Institute for Regenerative Medicine and Oral Implantology (UIRMI), 01007 Vitoria, Spain; roberto.tierno@bti-implant.es (R.T.); mohammad.hamdan@bti-implant.es (M.H.A.); 2 BTI-Biotechnology Institute, 01005 Vitoria, Spain; 3 Proteomics Platform, CIC bioGUNE, Basque Research and Technology Alliance (BRTA), CIBERehd, 48160 Derio, Spain; mazkargorta@cicbiogune.es (M.A.); felortza@cicbiogune.es (F.E.)

**Keywords:** atopic dermatitis, psoriasis, lichen sclerosus, platelet-rich plasma, skin inflammation, proteome, thermal inactivation

## Abstract

Atopic dermatitis, psoriasis and lichen sclerosus are among the most challenging conditions treated by dermatologists worldwide, with potentially significant physical, social and psychological impacts. Emerging evidence suggests that autologous-platelet-rich plasma could be used to manage skin inflammation. However, the presence of soluble autoimmune components could hinder their therapeutic potential. The aim of this study was to analyze the proteomic profile of plasma rich in growth factors (PRGFs) obtained from donors with inflammatory skin conditions to evaluate the impact of skin health status on the composition and bioactivity of PRGF-based treatments. Venous blood from healthy volunteers and patients with psoriasis, lichen sclerosus and atopic dermatitis was processed to produce PRGF supernatant. Half of the samples were subjected to an additional thermal treatment (56 °C) to inactivate inflammatory and immune molecules. Proteomic analysis was performed to assess the protein profile of PRGFs from healthy and non-healthy patients and the effect of Immunosafe treatment. Differential abundance patterns of several proteins related to key biological processes have been identified, including complement activation, blood coagulation, and glycolysis- and gluconeogenesis-related genes. These results also demonstrate that the thermal treatment (Immunosafe) contributes to the inactivation of the complement system and, as a consequence, reduction in the immunogenic potential of PRGF products.

## 1. Introduction

Inflammatory disorders represent the largest class of chronic skin diseases, affecting an estimated 20–25% of the population. These pathologies represent a major medical burden due to their high and increasing prevalence, potentially important effect on quality of life, absence of curative treatments, diagnostic challenges, co-morbidity, and significant economic impact [1]. Among the multiple types of skin inflammatory conditions, atopic dermatitis, psoriasis and lichen sclerosus are well-known and widespread chronic cutaneous disorders [2]. To reduce their negative impact on patients’ quality of life, numerous systemic treatment modalities have recently been introduced, specifically for severe types of these diseases. Biologic therapies target specific, dysregulated immune pathways in each pathology, including interleukin 4 receptor (IL4Rα) or Janus kinase 1 (JAK1) inhibitors in atopic dermatitis, interleukin 17 (IL-17) or 23 (IL-23) antagonists in psoriasis, and interleukin 4 (IL-4) and 13 (IL-13) signaling blockers in lichen sclerosus [3,4], representing significant advances in their treatment. However, approximately 20–50% of patients do not achieve significant symptom improvements even under this scenario [5,6,7].

The beneficial effect of PRP on a wide range of inflammation-related disorders has led to the assumption that PRP-based therapies may also be useful for treating chronic inflammatory skin conditions, including atopic dermatitis, psoriasis or lichen sclerosus [8]. Platelet-rich plasma (PRP) has emerged as a promising treatment modality in aesthetic dermatology, trichology, and wound management [9]. PRP is a biological product defined as a portion of the plasma fraction of autologous blood obtained by centrifugation that is enriched in platelets, along with a range of growth factors, chemokines, cytokines, and other plasma proteins [10]. As highlighted by Huber et al. [11], the intrinsic properties of PRP indicate that this hemoderivative can be used as an immunomodulator agent due to the wide range of secreted cytokines, particularly through the participation of Transforming Growth Factor β1 (TGF-β1) in the differentiation of T regulatory cells (Treg). In this sense, the scientific evidence regarding the efficacy of PRP-based therapies for treating inflammatory skin conditions is promising. As observed by Vafaei-Nodeh et al. [12], long-term control of atopic dermatitis can be successfully achieved via PRP therapy. Yosef et al. [13] also concluded that PRP combined with narrowband-ultraviolet B (NB-UVB) can be considered as a simple, acceptable, predictable and economical method for managing atopic dermatitis. In the same line, Lin et al. [14] highlighted the prophylactic effects of a xenogeneic PRP lotion on acute radiation dermatitis (ARD). After treating a total of 40 patients for inflammatory skin diseases with PRP, Kauhl et al. [8] reported that the average lesion size decreased and 50% of all patients achieved complete remission 53 months later. Regarding the management of psoriasis, Chakravdhanula et al. [15] evaluated the clinical efficacy of PRP in combination with methotrexate (MTX) in chronic plaque psoriatic patients. Their results revealed that patients treated with PRP/MTX showed a significant reduction in erythema, induration and desquamation at each visit, and psoriasis remitted after 16 weeks. Similar results have been obtained using PRP as a monotherapy for the treatment of plaque psoriasis [8,16,17,18]. According to Villalpando et al. [19], PRP may also be used as symptomatic coadjuvant treatment of lichen sclerosus. In this sense, PRP decreased histopathologic inflammation in women with vulvar lichen sclerosus without the potential side effects associated with topical or systemic immunomodulators [20]. Moreover, the combination of PRP and adipose-derived stem cell (ADSC) therapy has demonstrated significant improvements in lichen-sclerosus-related symptoms, including itching, burning, dyspareunia, and sexual function [21]. In the particular case of PRGFs, a specific subtype of PRP characterized by a moderate platelet concentration and the absence of leukocytes that is gaining interest in regenerative medicine because of its potential to stimulate and accelerate tissue healing and regeneration [22], a preliminary study suggests that this PRP subtype may be beneficial for treating inflammatory skin conditions, since PRGF formulations increased tissue viability and significantly reduced the overaccumulation of free radicals and cutaneous cytokine production in human organotypic skin explants. Case reports also evidenced a positive response after PRGF application in terms of skin quality improvement, local erythema decrease, and the amelioration of burning and itching [23].

Nevertheless, considering the autologous nature of PRP, the specific composition of blood could induce significant interindividual differences in terms of the biological effect and clinical efficacy of platelet-based treatments. The presence of soluble autoimmune components, such as immunoglobulins, complement proteins, inflammatory cytokines, or autoantibodies, in blood extracted from donors with inflammatory skin conditions could hinder their therapeutic potential [24,25,26,27]. Atopic dermatitis is characterized by the release of T-helper 2 (Th2) cytokines IL-4 and IL-13 and T-helper 17 (Th17) cytokines IL-17, IL-22, and interferon (IFN)-γ (chronic eczema) [28,29]. Psoriasis is characterized by IL-17, IL-22 and IL-23 [30,31]. Lichen sclerosus is related to interleukins 1 (IL-1) and 6 (IL-6) [32,33,34,35].

The local administration of these immunologic molecules on chronically affected areas of the skin may inhibit the anti-inflammatory effect of autologous growth factors and exacerbate the underlying pathology [36,37]. Hence, accurate proteomic characterization and optimization of PRP from pathological patients should be performed before considering its clinical use. The aim of this study was to analyze the proteomic profile of PRGF formulations obtained from donors with different inflammatory skin conditions and evaluate the impact of skin health status on their composition. The effect of a heat inactivation optimization step on proteomic composition was also assessed to reduce the immunologic content of PRGF samples.

## 2. Materials and Methods

This study was performed following the principles established in the Declaration of Helsinki amended in 2013 and in accordance with the ethical standards from the Araba University Hospital Clinical Research Ethical Committee (BTIIMD-01-IV-23-PRGF).

### 2.1. Preparation of PRGF Supernatants

Blood from donors with different inflammatory skin conditions was obtained. Three pathological groups were selected: patients with active psoriasis, patients with active lichen sclerosus and patients with active atopic dermatitis. An additional healthy donor group with no diagnosed skin condition was also included as a control. A total of three donors were included in each group. Of 12 patients, ages ranged from 15 to 73 years with a median of 41.6 years (IQR: 26.8), and 33% of the participants were male. Table 1 describes the data for each group in the study (Table 1).

PRGF-Endoret^®^ technology was used for the preparation of PRGF supernatant samples in sterile conditions following the manufacturer’s instructions (BTI Biotechnology Institute, Vitoria, Spain). After obtaining written informed consent, whole blood was withdrawn into a total of two 9 mL tubes containing 0.4 mL of 3.8% (*m*/*v*) sodium citrate as an anticoagulant. These tubes were centrifuged in a System V centrifuge (BTI) to separate blood components based on density gradient. The whole plasma column, avoiding the buffy coat, was pooled within each donor to obtain the plasma rich in growth factors (PRGFs). After that, PRGF samples were divided into two equal parts and transferred to individual tubes before the activation step in order to obtain two different formulations. One of these PRGF subsamples was activated with 10% *m*/*v* CaCl_2_ and incubated for 1 h at 37 °C (Plasmaterm H, BTI). Following the coagulation process, clot retraction led to the shrinking of the fibrin mesh driven by outside-in signaling by the platelet integrin complex αIIbβ3. At this point, the PRGF supernatant liberated during clot contraction was filtered (0.22 µm), aliquoted and stored at −80 °C [38]. This formulation was named PRGF supernatant (SP).

### 2.2. Immunosafe Treatment

The second PRGF subsample was subjected to a previously reported heat inactivation treatment (Immunosafe) as follows [39]: after obtaining PRGF supernatants, they were maintained for 45 min at 56 °C (Plasmaterm H plus, BTI). Finally, thermally treated PRGF supernatants were filtered, aliquoted and stored at −80 °C. This formulation was named PRGF Immunosafe-treated supernatant (INSP).

### 2.3. Proteomic Analysis

Protein was extracted by incubation in a buffer containing 7M urea, 2M thiourea, and 4% CHAPS for 30 min at RT under agitation and digested following the ilter-aided sample preparation protocol (FASP) described by Wiśniewski et al. [40] with minor modifications. Trypsin was added in 50mM ammonium bicarbonate to a trypsin:protein ratio of 1:10, and the mixture was incubated overnight at 37 °C. Peptides were dried out in an RVC2 25 SpeedVac concentrator (Christ, Osterode am Harz, Germany) and resuspended in 0.1% formic acid (FA). Peptides were desalted and resuspended in 0.1% FA using C18 stage tips (Millipore, Burlington, MA, USA) prior to acquisition. Digested samples were submitted to liquid chromatography–mass spectrometry (LC-MS) label-free analysis using a novel hybrid trapped ion mobility spectrometry quadrupole time of flight mass spectrometer (timsTOF Pro with PASEF, Bruker Daltonics, Billerica, MA, USA) coupled online to an Evosep ONE liquid chromatograph (Evosep, Odense, Denmark). A quantity of 200 ng was directly loaded onto the Evosep ONE and resolved using the 30-samples-per-day protocol.

Protein identification and quantification were carried out using MaxQuant software applying default settings [41]. Searches were performed against a database consisting of human protein entries (Uniprot/Swissprot), with precursor and fragment tolerances of 20 ppm and 0.05 Da. Only proteins identified with at least two peptides at FDR < 1% were considered for further analysis. Label-free quantification (LFQ) intensities were loaded onto Perseus Platform [42] and further processed (log2 transformation, imputation, etc.) before statistical analysis (Student’s t-test). A hierarchically clustered heatmap on the subset of differentially abundant proteins identified in at least one pairwise comparison (*p* ≤ 0.05) was constructed using the Euclidean distance and complete linkage method for clustering [43]. The R computing environment was used for proteomic data visualization [44]. The significance threshold for differential abundance analyses was set at *p* ≤ 0.05.

### 2.4. Functional Analyses

Gene Ontology (GO) enrichment analysis was carried out using the Database for Annotation, Visualization and Integrated Discovery (DAVID) online tool (http://david.abcc.ncifcrf.gov/summary.jsp, accessed on 8 January 2024) [45,46]. DAVID is an integrated biological knowledge base and analytic tool aimed at systematically extracting biologically relevant conclusions from large gene/protein datasets via GO and pathway enrichment analyses. Biological process (BP), cellular component (CC) and molecular function (MF) GO categories were assessed. The Kyoto Encyclopedia of Genes and Genomes (KEGG) pathway enrichment analysis was used for the functional analysis of the identified proteins. KEGG pathway is a database resource for understanding high-level functions and utilities of biological systems at different organization levels. A Fisher’s Exact test was used to determine whether the proportion of genes considered into certain terms or categories differ significantly between the dataset and the background. Only GO and KEGG terms enriched with a *p* ≤ 0.05 were considered for representation, comparison and discussion. Finally, GO term enrichment results were graphically represented through Sankey and dot plots using the SRPlot platform [47]. KEGG pathway maps were constructed via the PathView tool for pathway-based data integration and visualization [48]. The significance threshold for functional analyses was set at *p* ≤ 0.05.

## 3. Results

### 3.1. Effect of Skin Condition

**Figure 1:**
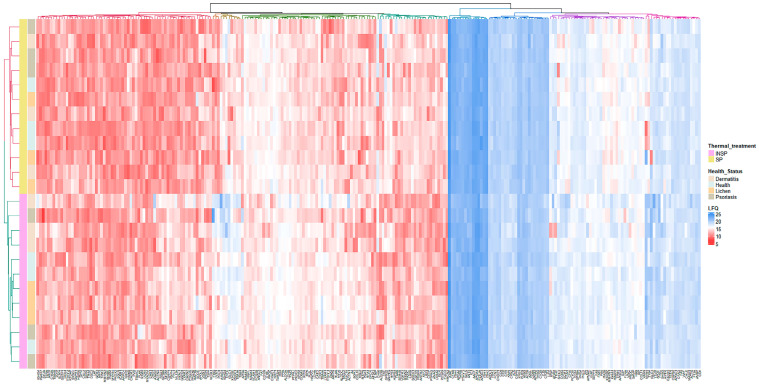
Proteomic heatmap with hierarchical clustering of MS-based label-free quantified proteins (LFQ) across the PRGF-supernatant-derived samples (SPs and INSPs) obtained from donors with different underlying health conditions: health, dermatitis, psoriasis, and lichen sclerosus. The protein list has been filtered to contain exclusively differentially abundant proteins identified in at least one pairwise comparison (*p* ≤ 0.05).

**Figure 2:**
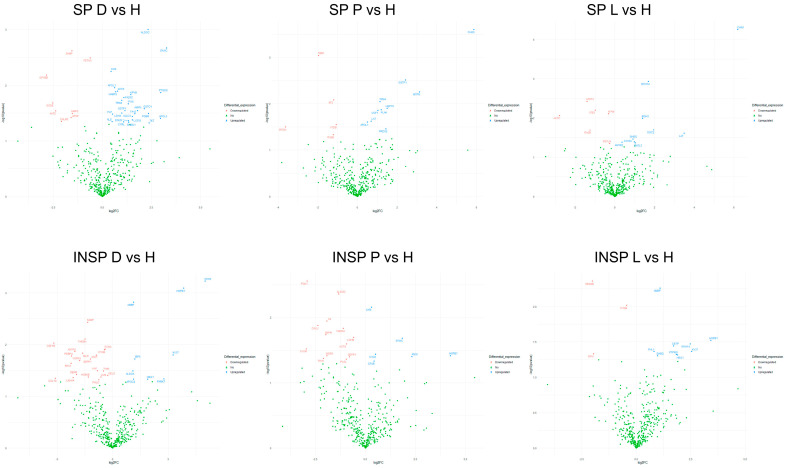
Volcano plots representing differentially abundant proteins in PRGF supernatants (SPs) and Immunosafe-treated PRGF supernatants (INSPs) between healthy (H) and pathological donors: dermatitis (D), psoriasis (P) and lichen sclerosus (L). The plots were constructed using log2 fold-change (log2FC) and *p* values. The colored dots represent the following cases: proteins upregulated in PRGF samples from pathological donors (blue), not differentially abundant proteins (green), and proteins downregulated in PRGF samples from pathological donors (red), at *p* ≤ 0.05.

The heatmap shown in Figure 1 illustrates the distribution of each differentially abundant protein and the clustering results. A clear difference between SP and INSP samples was detected, which revealed that the observed differences of thermal treatment on the proteomic profile were significant. Volcano plots representing the differential expression of proteins in PRGF supernatants from healthy and pathological donors are shown in Figure 2. Differential expression patterns between PRGF SP supernatants from healthy and dermatitis donors were identified for a total of 34 proteins (8.2%). When compared to healthy donors, supernatants obtained from donors with dermatitis showed higher expression levels of multiple-programmed-cell-death-, inflammation- and immune-cell-infiltration-related proteins, such as prostaglandin D2 synthase (PTGDS) [FC = 7.8; *p* = 0.001], fructose-bisphosphate aldolase C (ALDOC) [FC = 5.0; *p* = 0.001], tryptophanyl-tRNA synthetase (SYWC) [FC = 9.6; *p* = 0.002], apolipoprotein L1 (APOL1) [FC = 1.5; *p* = 0.01], peptidase inhibitor 16 (PI16) [FC = 2.5; *p* = 0.02], Tropomyosin alpha-4 (TPM4) [FC = 2.0; *p* = 0.02], apolipoprotein L3 (APOL3) [FC = 7.7; *p* = 0.04] and transketolase (TKT) [FC = 5.2; *p* = 0.05], or the complement component C6 (CO6) [FC = 1.4; *p* = 0.006], whereas the key antioxidant extracellular superoxide dismutase [Cu-Zn] (SODE) [FC = 0.17; *p* = 0.02] and the acute phase reactant serum amyloid P-component (SAMP) [FC = 0.34; *p* = 0.002], which activates immune cells and induces cytokines and chemokine, were downregulated in dermatitis donors. Differentially expressed proteins also included certain insulin-biosynthesis-and-secretion- and insulin-resistance-related polypeptides, including Fetuin A (FETUA) [FC = 0.65; *p* = 0.003], glutathione S-transferase P (GSTP1) [FC = 2.2; *p* = 0.03], L-lactate dehydrogenase A (LDHA) [FC = 0.35; *p* = 0.03], glutathione S-transferase omega-1 (GSTO1) [FC = 4.4; *p* = 0.03], or insulin-like growth factor-binding protein complex acid labile subunit (IGFALS) [FC = 1.4; *p* = 0.04], along with other proteins involved in blood clotting: platelet glycoprotein Ib (GP1BB) [FC = 0.14; *p* = 0.007], coagulation factor XII (F12) [FC = 3.1; *p* = 0.03], or coagulation factor VII (F7) [FC = 1.4; *p* = 0.03]. When compared to healthy donors, the proteomic profile of PRGF SP supernatants from donors with psoriasis and lichen revealed more similarities (with 15 differentially abundant polypeptides, yielding 3.6% of total proteins). The linker for the activation of T-cells (LAT), which is involved in the T-cell antigen receptor signal transduction pathway, and 60 kDa heat shock protein (CH60), a signaling molecule in the innate immune system, were overexpressed in supernatants from both psoriasis [FC = 1.6; *p* = 0.02 and FC = 58; *p* = 0.0002, respectively] and lichen donors [FC = 11; *p* = 0.02 and FC = 73; *p* = 5.6E05, respectively]. Conversely, integrin alpha-IIb (ITA2B), with a crucial role in the blood coagulation system, was downregulated in samples obtained from individuals with these health conditions [FC = 43; *p* = 0.05 and FC = 0.42; *p* = 0.02, respectively]. The list of differentially expressed proteins also includes immune-related molecules, such as in TPM4 [FC = 2.2; *p* = 0.009], Serglycin (SRGN) [FC = 0.08; *p* = 0.03] or antioxidant proteins, such as GSTP1 [FC = 5.3; *p* = 0.03] in psoriasis. Significant changes were also detected in several proteins implicated in cell adhesion as well as cell-surface-mediated signaling, including Integrin beta-3 (ITB3) [FC = 0.50; *p* = 0.006], transforming growth factor-beta-induced protein IG-H3 (BGH3) [FC = 2.6; *p* = 0.001] and Desmocollin-2 (DSC2) [FC = 3.9; *p* = 0.001] in donors with lichen, or 4F2 cell-surface antigen (4F2) [FC = 0.44; *p* = 0.008] and myotrophin (MTPN) [FC = 8.8; *p* = 0.005] in individuals with psoriasis.

The proteomic profile of PRGF supernatants was strongly affected by the Immunosafe treatment. The percentage of differentially abundant proteins in relation to healthy donors was 7.2% (30 proteins) in dermatitis, 4.6% in psoriasis (19 proteins) and 2.9% in lichen (12 proteins). INSP samples from donors with dermatitis showed significantly higher concentrations of CH60 [FC = 32; *p* = 0.0006], heat shock protein beta-1 (HSPB1) [FC = 14; *p* = 0.0008] and alpha-1-microglobulin/bikunin precursor (AMBP) [FC = 2.2; *p* = 0.001], an antioxidant and tissue repair protein with reductase, heme-binding and radical-scavenging activities, and lower levels of the target of Nesh-SH3 (TARSH) [FC = 0.35; *p* = 0.008], SAMP [FC = 0.39; *p* = 0.004], macrophage colony-stimulating factor 1 receptor (CSF1R) [FC = 0.11; *p* = 0.009], or the complement C1q subcomponent subunit B (C1QB) [FC = 0.74; *p* = 0.01] than those obtained from healthy donors. Similarly, when compared to samples derived from healthy individuals, INSP PRGF supernatants from lichen donors were also characterized by higher abundances of AMBP [FC = 2.1; *p* = 0.005] and HSPB1 [FC = 11; *p* = 0.03], but lower abundances of Semaphorin-4B (SEM4B) [FC = 0.24; *p* = 0.004] or C1QB [FC = 0.73; *p* = 0.01]. By contrast, the observed changes in protein abundance between PRGF supernatants from psoriasis and healthy donors subjected to heat treatments affected, among others, the complement C1r subcomponent (C1R) [FC = 1.2; *p* = 0.007], a protein implicated in glycolysis and the tricarboxylic acid cycle, the interferon-gamma-inducible cytoplasmic form of tryptophanyl-tRNA synthetase (SYWC) [FC = 3.6; *p* = 0.02], phosphoglycerate kinase 1 (PGK1) [FC = 0.13; *p* = 0.003], ALDOC [FC = 0.40; *p* = 0.004], the amyloid-beta precursor A4 (A4) [FC = 0.26; *p* = 0.01], or the complement C1q subcomponent A (C1QA) [FC = 1.4; *p* = 0.04].

**Figure 3:**
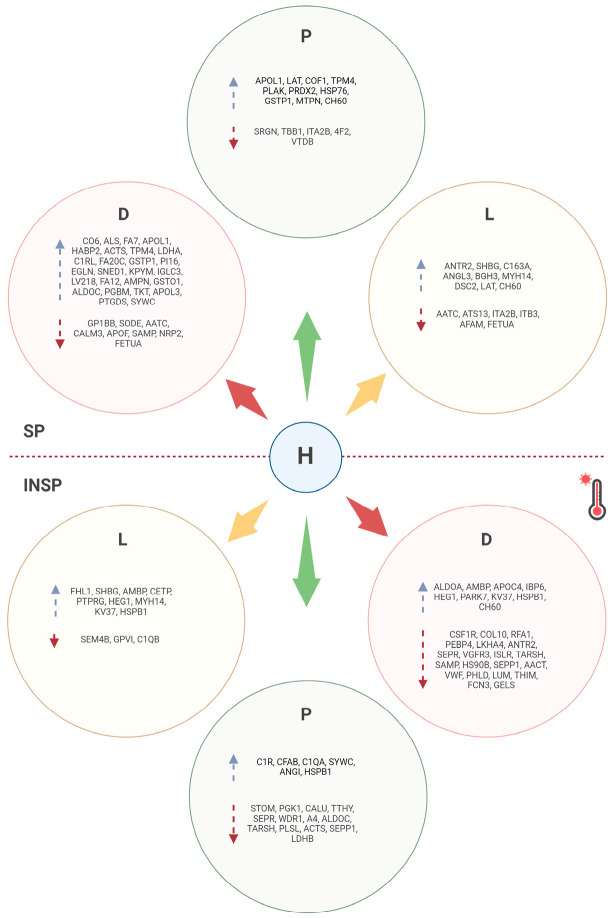
Schematic summary of differential protein expression between healthy and pathological donors as revealed by quantitative proteomic profiling.

A schematic summary of differential protein expression between healthy and pathological donors is depicted in Figure 3.

**Figure 4:**
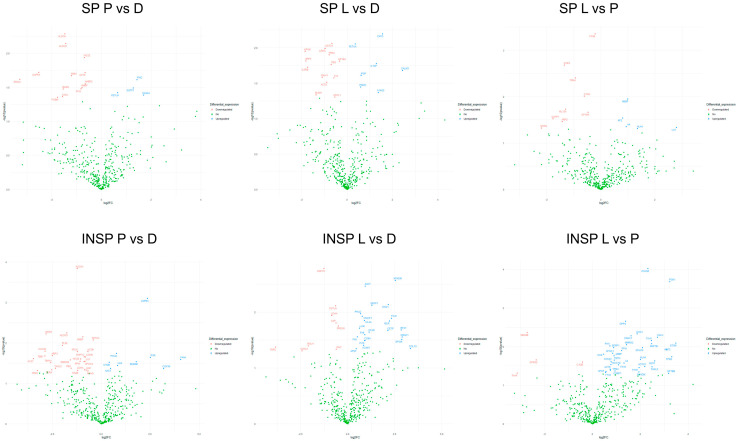
Volcano plots represent differentially abundant proteins in PRGF supernatants (SPs) and Immunosafe-treated PRGF supernatants (INSPs) between pathological donors: dermatitis (D), psoriasis (P) and lichen sclerosus (L). The plots were constructed using log2 fold-change (log2FC) and *p* values. The colored dots represent the following cases: proteins upregulated in PRGF samples from the following pathological donors: P, L and L, respectively (blue), not differentially abundant proteins (green), and proteins downregulated in PRGF samples from the following pathological donors: P, L, and L, in each case (red).

Results derived from pairwise comparisons between pathological disorders are provided in Figure 4. The proportion of differentially abundant proteins between PRGF supernatants from pathological donors was 3.4% (a total of 14 differentially abundant proteins between psoriasis and lichen donors), 4.1% (a total of 17 differentially abundant proteins between dermatitis and psoriasis donors) and 4.8% (a total of 20 differentially abundant proteins between dermatitis and lichen donors). PRGF supernatants from donors with dermatitis show higher expression levels of FETUA than those of psoriasis [FC = 1.6; *p* = 0.04] or lichen donors [FC = 1.3; *p* = 0.009], whereas TPM4 was downregulated in samples from lichen donors with respect to dermatitis [FC = 0.58; *p* = 0.01] or psoriasis donors [FC = 0.52; *p* = 0.004]. On the other hand, GSTP1 was significantly higher in supernatants extracted from donors with psoriasis than in those from donors with dermatitis [FC = 2.4; *p* = 0.03] or lichen [FC = 3.4; *p* = 0.03].

Regarding pairwise comparisons between Immunosafe-treated samples from pathological donors, the proportion of differentially abundant proteins was 8.9% (a total of 37 differentially abundant proteins between dermatitis and psoriasis donors), 7.5% (a total of 31 differentially abundant proteins between dermatitis and lichen donors) and 9.7% (a total of 40 differentially abundant proteins between psoriasis and lichen donors) in each case. In fact, differential abundance patterns identified in Immunosafe-treated samples resemble those of the PRGF supernatants described above. Certain key metabolic and glycolytic enzymes, including fructose-bisphosphate aldolase A (ALDOA) [FC = 0.43; *p* = 0.0001], ALDOC [FC = 0.29; *p* = 0.006], actin (ACTS) [FC = 0.63; *p* = 0.02], or L-lactate dehydrogenase B (LDHB) [FC = 0.60; *p* = 0.02], together with WD repeat-containing protein (WDR1) [FC = 0.14; *p* = 0.006], FETUA [FC = 0.75; *p* = 0.008], tubulin beta-1 (TBB1) [FC = 0.14; *p* = 0.02] and actin beta (ACTB) [FC = 0.63; *p* = 0.02], or the linker for the activation of T cells (LAT) [FC = 0.56; *p* = 0.03], were inhibited in INSP supernatants from psoriasis donors with respect to dermatitis donors. On the contrary, vascular endothelial growth factor receptor 3 (VGFR3) [FC = 5.1; *p* = 0.0008], phosphatidylinositol-glycan-specific phospholipase (PHLD) [FC = 1.7; *p* = 0.002], the complement C1QB chain [FC = 1.3; *p* = 0.03], or fumarylacetoacetase (FAAA) [FC = 17; *p* = 0.03] were upregulated in psoriasis donors. When compared with supernatants from donors with dermatitis, higher levels of myeloid-associated differentiation marker (MYADM) [FC = 5.8; *p* = 0.003], alpha-1-antichymotrypsin (AACT) [FC = 1.9; *p* = 0.003], Dystroglycan (DAG1) [FC = 4.4; *p* = 0.007], Selenoprotein P (SEPP1) [FC = 2.4; *p* = 0.008], and coagulation factors IX (FA9) [FC = 1.4; *p* = 0.03] and V (FA5) [FC = 1.9; *p* = 0.03], along with lower concentrations of FETUA [FC = 0.64; *p* = 0.008], L-lactate dehydrogenase A (LDHA) [FC = 0.56; *p* = 0.01] and glyceraldehyde-3-phosphate dehydrogenase (G3P) [FC = 0.65; *p* = 0.02], were detected in lichen donors. To mention some examples, in relation to psoriasis, INSP supernatants from donors with lichen showed higher levels of DAG1 [FC = 3.6; *p* = 0.005], cartilage acidic protein 1 (CRAC1) [FC = 3.0; *p* = 0.005], Calumenin (CALU) [FC = 6.6; *p* = 0.006], erythrocyte band 7 integral membrane protein (STOM) [FC = 11; *p* = 0.008], RAS-related protein Rap-1b (RAP1B) [FC = 5.5; *p* = 0.008], the complement C9 (CO9) [FC = 1.4; *p* = 0.02], or coagulation factors X (FA10) [FC = 2.1; *p* = 0.03] and XIII (F13A) [FC = 1.5; *p* = 0.05]. Some glycolysis- and gluconeogenesis-related enzymes, including phosphoglycerate kinase 1 (PGK1) [FC = 9.4; *p* = 0.0003], dipeptidyl peptidase 4 (DPP4) [FC = 2.6; *p* = 0.002] or transthyretin (TTHY) [FC = 5.5; *p* = 0.007], were also more abundant in supernatants obtained from donors with lichen. On the contrary, SEM4B [FC = 0.15; *p* = 0.004], G-protein coupled receptor 22 (GPR22) [FC = 0.20; *p* = 0.02] and the complement C1QB chain [FC = 0.75; *p* = 0.03] were significantly reduced under this pathological condition.

**Figure 5:**
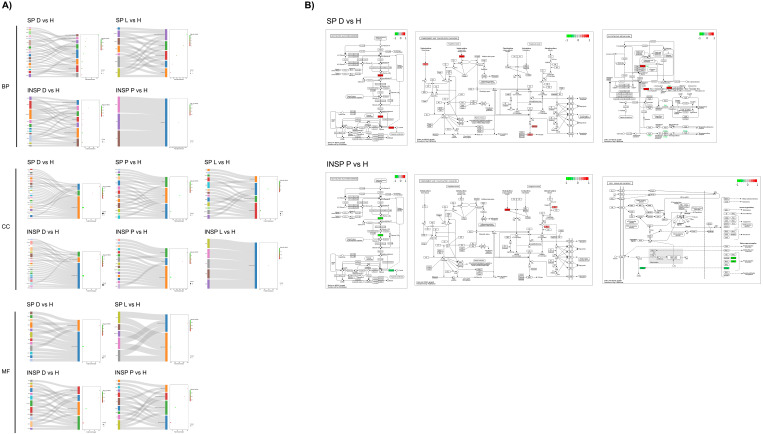
Functional analyses of differentially expressed genes (DEGs) between PRGF supernatants (SPs) and Immunosafe-treated PRGF supernatants (INSPs) obtained from donors including those with dermatitis (D), psoriasis (P) and lichen sclerosus (L) disorders and healthy donors (H). (**A**) Sankey dots diagrams representing Gene Ontology (GO) enrichment analyses, and (**B**) Kyoto Encyclopedia of Genes and Genomes (KEGG) pathway enrichment maps. Differentially expressed gene products are colored in red (upregulated in pathological supernatants) or green (downregulated in pathological supernatants). GO and KEGG enrichment analyses of DEGs were performed using DAVID. Only significantly enriched KEGG pathways and GO terms in biological process (BP), cellular component (CC), and molecular function (MF) branches are presented (*p* ≤ 0.05). All the statistically significant *p* values of the terms were negative 10-base log transformed.

As shown in Figure 5, Gene Ontology (GO) enrichment analyses of the differentially expressed proteins between dermatitis and healthy donors in SP supernatants revealed that many significantly enriched GO terms were related to the extracellular space (*p* = 1.1 × 10^−13^), blood microparticles (*p* = 4.2 × 10^−6^), and vesicles (*p* = 4.2 × 10^−6^). In addition, the integrin-mediated signaling pathway (*p* = 6.9 × 10^−7^), cell matrix adhesion (*p* = 4.7 × 10^−5^), and extracellular matrix organization processes (*p* = 2.4 × 10^−4^) were altered in SP samples from donors with lichen. The components cell surface (*p* = 5.1 × 10^−5^) and extracellular region (*p* = 2.8 × 10^−4^) were predominantly affected by changes in protein abundance patterns between donors with lichen and healthy individuals.

**Figure 6:**
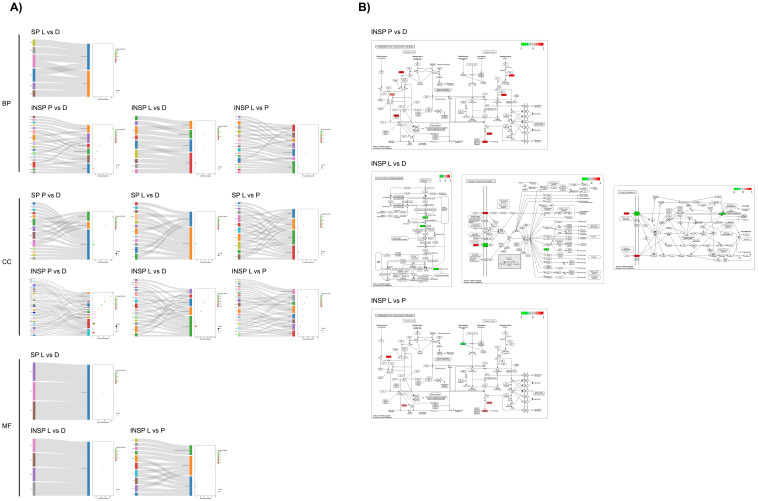
Functional analyses of differentially expressed genes (DEGs) in PRGF supernatants (SPs) and Immunosafe-treated PRGF supernatants (INSPs) between pathological donors: dermatitis (D), psoriasis (P) and lichen sclerosus (L). (**A**) Sankey dot diagrams representing Gene Ontology (GO) enrichment analyses, and (**B**) Kyoto Encyclopedia of Genes and Genomes (KEGG) pathway enrichment maps. Differentially expressed gene products are colored in red (upregulated in P, L, and L, respectively) or green (downregulated in P, L, and L, in each case). GO and KEGG enrichment analyses of DEGs were retrieved using DAVID. Only significantly enriched KEGG pathways and GO terms in biological process (BP), cellular component (CC), and molecular function (MF) branches are presented (*p* ≤ 0.05). All the statistically significant *p* values of the terms were negative 10-base log transformed.

Similarly, when comparing pathological conditions, GO enrichment analyses revealed that differences in PRGF SP samples between dermatitis and lichen donors were limited to blood coagulation processes (*p* = 9.7 × 10^−5^), affecting several proteins associated with the extracellular space (*p* = 2.8 × 10^−6^) and the external side of the plasma membrane (*p* = 8.7 × 10^−4^), and in the extracellular region (*p* value = 2.6 × 10^−9^) together with the secretory granule lumen (*p* = 9.2 × 10^−5^) after testing dermatitis vs. psoriasis (Figure 6). On the other hand, the comparison of psoriasis vs. lichen revealed significant differences in proteins associated with the plasma membrane (*p* = 3.3 × 10^−2^). In the same line, INSP samples obtained from donors with dermatitis showed higher levels of proteins implicated in the glycotytic process (*p* = 1.9 × 10^−6^), platelet aggregation (*p* = 2.4 × 10^−6^), microtubule cytoskeleton organization (*p* = 0.003), and cell adhesion (*p* = 0.02) than heat-inactivated supernatants from psoriasis donors. Among the most commonly affected cell components were the secretory granule lumen (*p* = 5.6 × 10^−8^), the actin cytoskeleton (*p* = 4.1 × 10^−7^), the cytosol (*p* = 3.9 × 10^−5^), stress fibers (*p* = 0.008), the plasma membrane (*p* = 0.02), and the cell surface (*p* = 0.03). When comparing dermatitis- vs. lichen-derived INSP samples, differentially abundant proteins were involved in proteolysis (*p* = 0.03), blood coagulation (*p* = 0.009) and the glycolytic process (*p* = 0.002). Cellular locations of these gene products include the plasma membrane (*p* = 0.03), vesicles (*p* = 0.002), the perinuclear region of cytoplasm (*p* = 0.004) and the external side of plasma membrane (*p* = 0.005). Considering the comparison between INSP samples from psoriasis and lichen donors, the differentially abundant proteins were enriched in the cholesterol metabolic process (*p* = 0.0005), cell adhesion (*p* = 0.0006), active filament organization (*p* = 0.003), lipid transport (*p* = 0.01), or blood coagulation biological processes (*p* = 0.01), together with different cellular components, including actin filaments (*p* = 5.6 × 10^−8^), blood microparticles (*p* = 7.3 × 10^−6^), stress fibers (*p* = 9.7 × 10^−6^), high-density lipoprotein particles (*p* = 1.6 × 10^−5^), or the cytoskeleton (*p* = 0.02).

### 3.2. Effect of the Immunosafe Treatment

**Figure 7:**
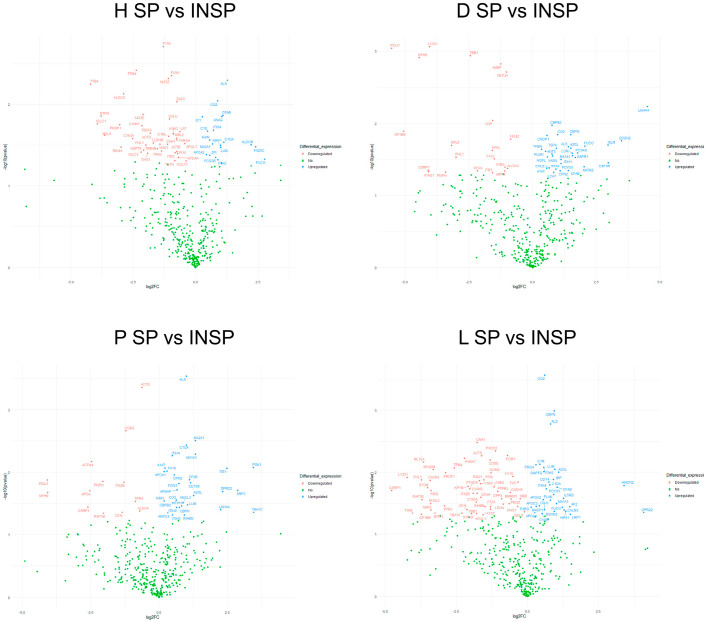
Volcano plots representing differentially abundant proteins between PRGF supernatants (SPs) and Immunosafe-treated PRGF supernatants (INSPs) within health condition: healthy (H), dermatitis (D), psoriasis (P) and lichen sclerosus (L). The plots were constructed using log2 fold-change (log2FC) and *p* values. The colored dots represent the following cases: proteins upregulated in SP samples (blue), not differentially abundant proteins (green), and proteins downregulated in SP samples (red), at *p* ≤ 0.05.

As depicted in Figure 7, the effect of thermal treatment on protein abundance was not homogeneous across health conditions. The percent of total proteins that showed significant differential abundances between PRGFs and thermally treated PRGF supernatants reached 11% in samples from donors with psoriasis, 12% in donors with dermatitis, 14% in healthy donors and 21% in donors with lichen. Thermal treatment significantly reduced the concentration of IGFALS [FC = 0.41; *p* = 0.03 in healthy donors, FC = 0.38; *p* = 0.02 in donors with dermatitis, FC = 0.50; *p* = 0.0003 in donors with psoriasis, and FC = 0.57; *p* = 0.002 in donors with lichen], complement factor B (CFAB) [FC = 0.47; *p* = 0.01 in healthy donors, FC = 0.38; *p* = 0.05 in donors with dermatitis, FC = 0.46; *p* = 0.01 in donors with psoriasis, and FC = 0.49; *p* = 0.02 in donors with lichen], Lumican (LUM) [FC = 0.50; *p* = 0.03 in healthy donors, FC = 0.34; *p* = 0.03 in donors with dermatitis, FC = 0.49; *p* = 0.03 in donors with psoriasis, and FC = 0.51; *p* = 0.009 in donors with lichen] and complement C2 (CO2) [FC = 0.54; *p* = 0.009 in healthy donors, FC = 0.51; *p* = 0.001 in donors with dermatitis, FC = 0.65; *p* = 0.03 in donors with psoriasis, and FC = 0.65; *p* = 0.003 in donors with lichen] in the PRGF supernatant regardless of the health condition of the donor. In addition, the Immunosafe treatment led to higher levels of F13A [FC = 2.5; *p* = 0.002], Filamin-A (FLNA) [FC = 2.0; *p* = 0.004], thymosin beta-4 (TYB4) [FC = 18.4; *p* = 0.006], ALDOC [FC = 7.4; *p* = 0.007], 14-3-3 protein zeta/delta (1433Z) [FC = 2.1; *p* = 0.005], 14-3-3 protein epsilon (1433E) [FC = 4.3; *p* = 0.01] or MTPN [FC = 13.8; *p* = 0.01] in healthy individuals. A similar trend was observed for carboxypeptidase N (CBPN) [FC = 0.35; *p* = 0.01 in donors with dermatitis, FC = 0.60; *p* = 0.04 in donors with psoriasis, and FC = 0.52; *p* = 0.001 in donors with lichen] in pathological samples, whereas integrin beta-3 (ITB3) [FC = 3.0; *p* = 0.05 in donors with dermatitis, and FC = 3.1; *p* = 0.02 in donors with lichen] and 1433Z [FC = 1.8; *p* = 0.02 in donors with dermatitis, and FC = 2.5; *p* = 0.04 in donors with lichen] increased in Immunosafe-treated supernatants with the exception of those from donors with psoriasis. Inter-alpha-trypsin inhibitor heavy-chain ITIH2 and ITIH4 also decreased in Immunosafe-treated supernatant samples regardless of health status: healthy donors [FC = 0.54; *p* = 0.05, and FC = 0.61; *p* = 0.02, respectively], donors with dermatitis [differential abundances were only significant in ITIH4; FC = 0.53; *p* = 0.04], psoriasis [FC = 0.65; *p* = 0.04, and FC = 0.72; *p* = 0.005, respectively] and lichen [FC = 0.67; *p* = 0.008, and FC = 0.63; *p* = 0.02, respectively].

**Figure 8:**
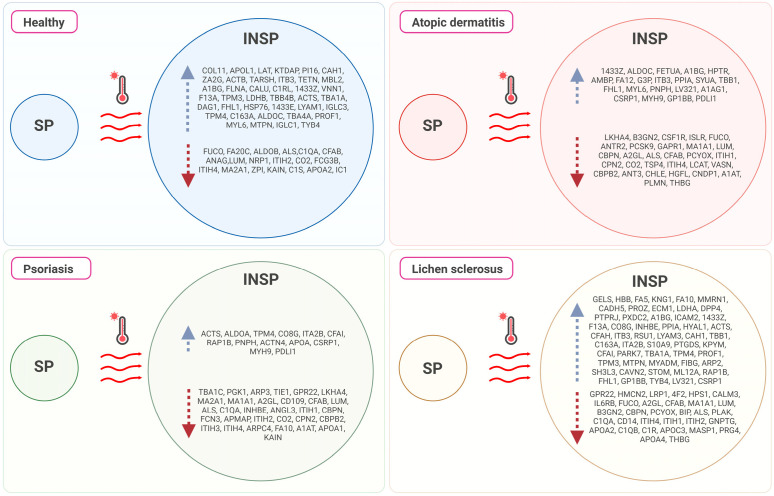
Schematic summary of the effect of Immunosafe treatment on differential protein abundance of PRGF supernatants as revealed by quantitative proteomic profiling.

A schematic summary of differential protein abundance between SP and INSP samples is presented in Figure 8.

**Figure 9:**
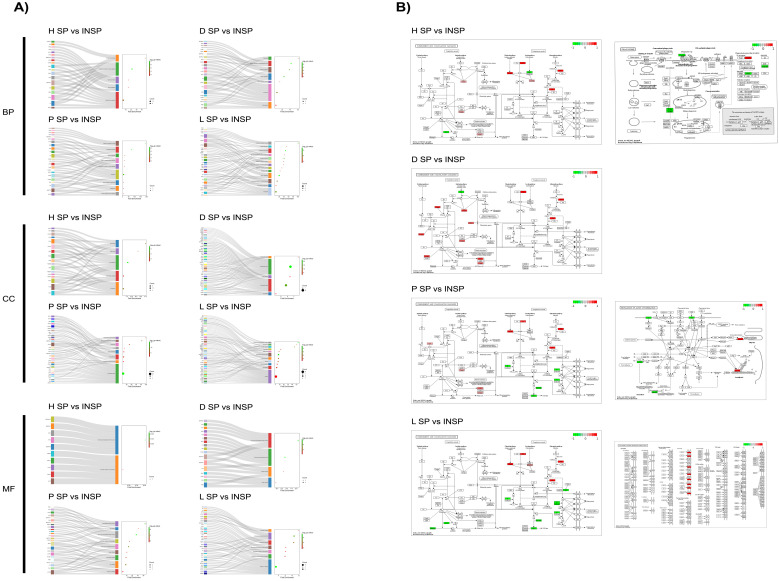
Functional analyses of differentially abundant gene products between PRGF supernatants (SPs) and Immunosafe-treated PRGF supernatants (INSPs) within health condition: healthy (H), dermatitis (D), psoriasis (P) and lichen sclerosus (L). (**A**) Sankey dot diagrams representing Gene Ontology (GO) enrichment analyses, and (**B**) Kyoto Encyclopedia of Genes and Genomes (KEGG) pathway enrichment maps. Differentially expressed gene products are colored in red (upregulated in SP) or green (downregulated in SP). GO and KEGG enrichment analyses of differentially abundant gene products were retrieved using DAVID. Only significantly enriched KEGG pathways and GO terms in biological process (BP), cellular component (CC), and molecular function (MF) branches are presented (*p* ≤ 0.05). All the statistically significant *p* values of the terms were negative 10-base log transformed.

According to Figure 9, GO enrichment analyses of the differentially abundant proteins confirmed that the impact of thermal treatment on the proteomic profile of PRGF supernatants is particularly significant in blood-coagulation-related proteins regardless of the health status of the donors (*p* = 0.002 in healthy donors, *p* = 4.0 × 10^−6^ in dermatitis donors, *p* = 0.001 in psoriasis donors, and *p* = 1.5 × 10^−7^ in lichen donors). Lower levels of complement-activation-related proteins were also identified in Immunosafe-treated supernatants from healthy (*p* = 7.8 × 10^−5^), psoriasis (*p* = 5.2 × 10^−7^) and lichen donors (*p* = 1.5 × 10^−7^), while thermal treatment inhibited several proteins involved in proteolytic processes exclusively in supernatants from pathological donors (*p* = 0.0002 in dermatitis donors, *p* = 7.5 × 10^−6^ in psoriasis donors, and *p* = 2.8 × 10^−5^ in lichen donors). Depending on the pathology, a significant effect of thermal treatment on the hyaluronan metabolic process was observed (*p* = 9.0 × 10^−7^ in psoriasis donors, and *p* = 6.4 × 10^−6^ in lichen donors). Certain complex biomolecules and structures were also significantly affected by thermal treatment, including focal adhesion (*p* = 3.2 × 10^−6^ in healthy donors, *p* = 0.0007 in dermatitis donors, and *p* = 0.0003 in psoriasis donors), extracellular region (*p* = 9.9 × 10^−23^ in dermatitis donors, and *p* = 5.4 × 10^−16^ in dermatitis donors), cytoskeleton (*p* = 0.0002 in dermatitis donors, and *p* = 0.009 in lichen donors), and actin cytoskeleton (*p* = 0.002 in psoriasis donors, and *p* = 0.03 in lichen donors). Impaired serine-type endopeptidase inhibitor activities (*p* = 0.0003 in healthy donors, *p* = 8.5 × 10^−6^ in dermatitis donors, and *p* = 0.01 in lichen donors), serinE-type endopeptidase activities (*p* = 8.3 × 10^−6^ in dermatitis donors, *p* = 7.0 × 10^−5^ in psoriasis donors, and *p* = 1.9 × 10^−6^ in lichen donors) and structural constituents of cytoskeleton (*p* = 0.0003 in healthy donors, *p* = 0.002 in psoriasis donors, and *p* = 0.01 in lichen donors) were observed in Immunosafe-treated supernatants. Other proteins involved in different molecular functions, including integrin binding (*p* = 0.008 in dermatitis donors, and *p* = 0.006 in psoriasis donors) and calcium ion binding (*p* = 0.03 in psoriasis donors, and *p* = 3.4 × 10^−6^ in lichen donors), were also altered.

## 4. Discussion

This study has shown the influence of immunoinflammatory diseases in the genetic expression profile of crucial biological processes like blood coagulation, complement activation, and glycolysis and gluconeogenesis. Moreover, heat treatment of PRGFs has led to the inhibition of the complement system and, consequently, to a reduction in the immunogenic potential of PRGF-derived formulations.

Chronic inflammatory diseases have influenced the proteomic profile of PRGF supernatants (as compared to healthy patients). Atopic dermatitis has activated anaerobic glycolysis, the complement system, coagulation and glutathione metabolism. Other studies have reported similar results [49,50,51,52,53]. Under anaerobic conditions, the cytosolic enzyme lactate dehydrogenase (LDH) converts pyruvate to lactate and regenerates NAD+ from NADH, producing 2 ATP per glucose molecule, thus providing a direct energy source in the absence of oxygen (anaerobic glycolysis). According to the present data, this action was modulated via upregulation of three key enzymes: fructose-bisphosphate aldolase, often called aldolase (ALDO), L-lactate dehydrogenase (LDH) and pyruvate kinase (KPYM). The ALDO enzyme is considered an agonist of key glycolytic enzymes that catalyze the conversion of fructose 1-6-diphosphate to glyceraldehyde 3-phosphate, a metabolite that occurs as an intermediate in both glycolysis and gluconeogenesis. Zhang et al. [54] also reported elevated ALDO in serum from patients diagnosed with moderate-to-severe atopic dermatitis. On the other hand, KPYM catalyzes the transfer of a phosphate group from phosphoenolpyruvate (PEP) to adenosine diphosphate (ADP), yielding one molecule of pyruvate and one molecule of ATP. Finally, the oxidoreductase LDH converts pyruvate to lactate with the reduction of NAD+ to NADH, and vice versa [55]. Unexpectedly, a significant relationship between dermatitis and the levels of certain insulin-biosynthesis-and-secretion- or insulin-resistance-related polypeptides (GSTO1, FETUA, GSTP1, IGFALS or LDHA) was observed. Even though insulin has an important role in homeostasis and physiology of the skin, the precise function of insulin signaling remains controversial. Under healthy conditions, insulin regulates the equilibrium between the proliferation and differentiation of keratinocytes, which could be considered a prerequisite for the correct formation of the epidermal structure. However, under chronic inflammatory conditions, high levels of proinflammatory cytokines activate p38MAPK, which induces insulin resistance by serine phosphorylation of insulin receptor substrates, inhibiting differentiation and, at the same time, increasing the proliferation rate of basal keratinocytes [56]. In particular, Savaş-Erdoğan et al. [57] identified the association between sebhorreic dermatitis and insulin resistance, whereas a glutathione S-transferase M1 (GSTM1) polymorphism was also associated with atopic dermatitis as revealed by Cho et al. [58]. As reviewed by Hu et al. [59], impaired insulin signaling and increased insulin resistance, as well as elevated status of chronic inflammation, are major risk factors that may induce skin diseases.

There is increasing evidence regarding the participation of the complement system and blood coagulation in the inflammatory processes associated with different types of dermatitis [60,61,62,63]. When compared with the healthy control, the classical pathway of the complement activation system has been upregulated in samples from individuals with skin disorders. The complement system plays a key role in the innate defense against pathogens. Its activation leads to robust and efficient proteolytic cascades, which terminate in opsonization and lysis of the pathogens, as well as in the generation of an inflammatory response through the production of strong proinflammatory molecules [64]. The classical complement pathway plays a role in both innate and adaptive immunity [65], and it is activated when the C1 complex binds, via the C1q subcomponent, to the Fc domain of antibody in immune complexes, to nonimmunoglobulin activators such as apoptotic cells, or to other polyanionic substances [66]. The membrane attack complex (MAC) and their components (complement proteins C6, C7, C8 and C9) have been increased in PRGF supernatant from patients with dermatitis. This complex is known as a cytolytic effector of innate and adaptative immunity that forms pores in the plasma membrane of pathogens or targeted cells, leading to osmolysis [67]. Dahl et al. [68] detected deposits of MAC in the dermal papillae of patients with dermatitis herpetiformis, which evidenced that the complement system was activated to the terminal complement sequence. Moreover, as revealed by the increased levels of factors VII (F7) and XII (F12), both the extrinsic and intrinsic coagulation pathways were activated in patients suffering from dermatitis. Accordingly, Cugno et al. [69] also found higher levels of plasmatic markers of thrombin generation and fibrinolysis, including prothrombin fragment F1+2, activated F7, thrombin-antithrombin complex and D-dimer, and fibrinogen/fibrin degradation products (FDP), in patients with chronic spontaneous urticaria, angioedema, and bullous pemphigoid. On the other hand, F12 is the principal initiator of the plasma contact system and has proinflammatory and prothrombotic activities. In fact, a strong relationship between IgE-mediated allergic responses and the local activation of the intrinsic plasma coagulation-kinin pathways has been highlighted in various atopic diseases [69,70,71].

Regarding glutathione metabolism, our data revealed higher expression of two key enzymes: aminopeptidase N (ANPEP) and glutathione S-transferase (GST). GST genes are upregulated in response to oxidative stress and their enzymatic products protect cells by inhibiting various pathways and redox-associated signal molecules [72]. In this sense, genetic polymorphisms affecting GST produce a decrease in the intracellular concentration of glutathione, with consequent raising of skin inflammation, as seen in atopic and allergic dermatitis [73,74,75,76]. Cho et al. [77] observed that GSTP1 was strongly expressed in the upper epidermis in the chronic stage of acute and chronic psoriasis or eczematous dermatitis. Coagulation factor XII (F12) is converted into F12a following contact with a negatively charged surface. F12a initiates the classical complement cascade and shows direct proinflammatory properties [78]. As reviewed by Didiasova et al. [79], the active form triggers the kallikrein–kinin system, leading to the release of bradykinin (BK) from high-molecular-weight kininogen (HK), launches the intrinsic blood coagulation pathway via activation of factor 11 (F11), initiates fibrinolysis via PKa-mediated urokinase activation, and activates C1 esterases, which constitute the first components of the macromolecular C1 complex from the classical complement pathway. Bradykinin is considered a strong histamine-independent pruritogen in lesional skin of atopic dermatitis [80], and the relationship between F12 and atopic dermatitis [81] or angioedema has been suggested [82,83]. According to René and Stavrou [84], F12 shows activities independent of its protease function, contributing to macrophage polarization, upregulating neutrophil functions and inducing T-cell differentiation.

The relationship between integrin induction, altered angiogenesis, vascular permeability, endothelial cell functionality and multiple inflammatory skin conditions has been widely reported in previous publications [85,86,87,88,89]. For example, a positive peroxidase reaction to aminopeptidase-N (APN/CD13) was observed on human keratinocytes from patients with chronic skin inflammation by Hunyadi et al. [90]. Among other biological functions, including the enzymatic cleavage of peptides, endocytosis, signal transduction, cellular differentiation, proliferation, apoptosis, motility and chemotaxis, antigen presentation, phagocytosis and cell adhesion, APN/CD13 participates in angiogenesis and is implicated in the process of capillary tube formation [91]. Other groups have reported that different environmental conditions and angiogenic factors induce CD13 expression in endothelial cells and that enzymatic activity inhibitors impair angiogenesis in vitro and in vivo [92,93]. The case of perlecan, a major heparan sulfate proteoglycan of vascularized tissues codified by the gene HSPG2, should also be mentioned. It has been demonstrated that extracellular matrix protein 1 (ECM-1), which is a soluble glycoprotein with promiscuous extracellular binding targets that includes basement proteins, perlecan, phospholipids and proteolytic enzymes, is responsible for the structural organization, integrity and function of human skin [94,95]. Data indicate that autoantibodies to ECM-1 are a common feature in certain chronic skin conditions, thus obstructing the usual regulatory binding of ECM1 to matrix metalloproteinase-9 (MMP9), which results in overreactive collagenase activity disrupting collagen homeostasis and, as a consequence, disruption of the focal basement membrane. In this sense, any alteration in the functional binding of ECM1 to MMP9, collagen IV or perlecan would result in pathology [96].

PRGFs is a source of a plethora of signaling molecules including cytokines, growth factors and lipids among others that exert versatile immunomodulatory actions, in the relative absence of any significant side effects. However, the presence of soluble autoimmune components in blood extracted from donors with inflammatory skin conditions necessitates the development of strategies to reduce its inflammatory components [25]. Thermal treatment to inactivate immunomodulatory molecules has significantly affected the proteomic profile of PRGF supernatants. Coagulation factor 13 (F13) has been also upregulated after thermal treatment in samples obtained from both healthy donors and donors with lichen planus, whereas the expression of fibrinogen increased in samples obtained from donors with lichen planus. Both are key proteins involved in clot formation producing a stable clot resistant to degradation, whereby fibrinogen provides the matrix that is modified by activated F13 (F13a), acting as a cross-linker. In its role as a transglutaminase, F13a not only cross-links fibrin chains for clot stabilization but also cross-links other plasma proteins involved in clot formation and fibrinolysis to fibrin [97]. According to the present data, thermal treatment also tended to increase factor I (FI) expression in samples obtained from patients with psoriasis and lichen planus. FI is a protein of the complement system that regulates complement activation by cleaving C3b and C4b, leading to downregulation of the construction of C3 convertase and the inhibition of the complement cascade. Other important regulators of the complement system, including mannose-binding protein (MBP), factor 12 (F12) and factor H (FH), were also overexpressed following thermal treatment under certain health conditions. The FH component acts as a cofactor for the proteolytic inactivation of C3b by FI and accelerates the decay of the C3bBb convertase produced in the alternative pathway by displacing the Bb binding partner to C3b to make a new complex with C3b, thus ceasing further generation of C3b [98,99].

On the other hand, thermal inactivation significantly reduced complement C2 concentrations, a serum glycoprotein that is necessary for the formation of C3 convertase, the key enzyme of the classical pathway of complement activation [100]. This multi-domain serine protease, which is cleaved by activated factor C1 into two fragments (C2b and C2a), provides catalytic activity to the C3 and C5 convertases of classical and lectin pathways of complements [101]. C1qrs complex activation, which constitutes the first component of the classical complement pathway, was also inhibited in Immunosafe-treated samples. Active C1 splits complement C4 and C2, releasing C4a, C4b, C2a and C2b small proteins and stimulating the creation of the classical pathway C3 convertase (C4bC2b complex) that promotes the cleavage of C3 [102]. Conversion of native C3 to a C3b-like form resulting from the spontaneous hydrolysis of the thioester in C3 conforms to the initial C3 convertase. Thus, C3(H_2_O) binds FB in a Mg^2+^ stabilized complex, C3(H_2_O)Bb. The Bb fragment expresses serine protease activity but can cleave C3 and C5 only while it remains bound to C3b [103]. Moreover, factor B (FB) was also downregulated following thermal inactivation. One of the key roles of FB is in the initiation of the alternative pathway of complement activation. It contains a serine protease domain and, when activated, it provides the catalytic activity of the alternative pathway C3 and C5 convertases [104]. Factor B is functionally similar to the classical pathway component C2. It is generated as a single-chain glycosylated protein, and cleavage by factor D generates two peptide fragments (Ba and Bb). The Bb fragment expresses serine protease activity but can cleave C3 and C5 only while it remains bound to C3(H2O) and C3b, thereby constituting Mg^2+^ stabilized complexes usually known as alternative pathway convertases (C3(H_2_O)Bb and C3bBb) [105]. As part of the C3 convertases, the serine protease domain of Bb has specific catalytic activity for the cleavage of C3 molecules. The addition of another C3b molecule to the alternative pathway C3 convertase generates C5 convertase (C3bBbC3b). Regarding the alternative pathway C5 convertase, the serine protease domain of Bb cleaves C5 molecules, enabling the assembly of C5-C9 and the resultant formation of the membrane attack complex [101,106].

Circulating plasminogen (PLG), a glycoprotein that was reduced after thermal treatment of samples extracted from donors with dermatitis, is another regulator of complement with multiple physiological roles as an enzyme that is capable of breaking down different tissue barriers and promoting fibrinolysis. Regarding the complement system, PLG regulates the classical and lectin pathways and also plays a minor role in modulating the alternative pathway. PLG can be converted into its active form, plasmin, by several proteases originating from the host or from invading microorganisms. Afterwards, plasmin is capable of binding either C3 or C5 and cleaving them into inactive forms through the action of its serine protease domains [107]. It should be considered that the complement-activated product C5a is a chemotactic agent that displays powerful biological activities since it is involved in different immune functions, including the recruitment of inflammatory cells and T lymphocytes, the activation of phagocytic cells, the release of granule-based enzymes and the generation of oxidants [108]. Moreover, plasmin can bind C3b, whereby it will increase the cleavage rate by factor I, resulting in inactive C3b regulating the alternative pathway [109]. Thermal treatment of samples also inhibited the expression of serpin—with the exception of samples derived from donors with lichen planus—and CPB2 in supernatants from patients with psoriasis and dermatitis. Serpins are members of the serine proteinase inhibitor superfamily that function as components of innate antiviral immunity, regulating the activity of serine proteases and playing key roles in blood coagulation, fibrosis, complement activation, fibrosis and inflammation. CPB2 removes C-terminal lysine residues from fibrin, leading to reduced incorporation of plasminogen and tissue plasminogen activator into the partially digested clot, resulting in the inhibition of fibrinolysis along with a reduction in prourokinase-mediated activation of plasminogen. More specifically, CPB2 removes C-terminal arginine residues from bradykinin and complements C3a and C5a, thereby inactivating them. The activation of proCPB2 by the thrombin/TM complex serves as a homeostatic negative feedback mechanism to dampen adverse effects of excessively generated pro-inflammatory mediators at sites of tissue injury [110,111].

The present study has two methodological limitations: the small number of participants and the observed heterogeneity in medication use patterns. In this sense, a suboptimal sample size could lead to reduced statistical power and a lack of reproducibility. A related issue may be the presence of confounders in the context of highly variable population groups with large subpopulations, with a particular emphasis on the potential effect of biological medications or anti-inflammatory drugs used in the treatment of chronic inflammatory skin disorders on the proteome of PRGFs. However, it should be considered that the present preliminary investigation was designed to identify and document the existence of an effect derived from differences in the skin health status or the implementation of a thermal treatment rather than quantifying the general performance within a population. Appropriate statistical approaches are selected to reach valid conclusions and reduce the impact of the limited sample size that this study has. Future research should focus on measuring the impact of different health conditions or PRGF preparation protocols on the proteomic profile of PRGF formulations using larger samples sizes and restricting the analyses by specific confounding variables.

In conclusion, differential gene expression profiles affecting crucial biological processes were detected after comparing PRGF supernatants from donors with varying health status, including blood coagulation, complement activation, and glycolysis- and gluconeogenesis-related genes. Moreover, despite some differences having been detected between samples obtained from donors with contrasting health conditions, thermal treatment contributes to the inhibition of the complement system and, consequently, to a reduction in the immunogenic potential of PRGF-derived formulations. Specifically, thermal treatment attenuates the complement cascade via downregulating the three known pathways of complement activation and, mainly, through the inhibition of C2 and C1 complex—early components of the complement system—and FB, a centrally important component of the alternative pathway. Complement activation products (CAPs) are considered pro-inflammatory mediators and biochemical markers of different inflammatory skin diseases [60,112,113]. Depending on the health condition of the donors, this effect may be accompanied by the upregulation of FI, a crucial inhibitor controlling all complement pathways due to its ability to degrade activated complement proteins C3b and C4b, late-acting complement proteins (C6, C7, C8 and C9) and the terminal complement complex (MAC). Under certain health conditions, other proteins are modulated by thermal treatment, including multiple serpines, F13, fibrinogen, CPB2 and PLG, thus potentially modulating different metabolic pathways, including the coagulation cascade, platelet activation and the complement system.

## Figures and Tables

**Table 1:** Age and sex of patients included in each study group.

Study Group	Mean Age (Years)	Sex (Number of Patients)	
		Male	Female
Healthy	51 (range: 33 to 69)	2	1
Atopic Dermatitis	28 (range: 15 to 52)	1	2
Psoriasis	50 (range: 36 to 65)	0	3
Lichen sclerosus	41 (range: 20 to 61)	0	3

## Data Availability

The data presented in this study are available on request from the corresponding author as they contain information that could compromise the privacy of research participants.
